# Crosstalk between autophagy-dependent ferroptosis and PANoptosis in myocardial and cerebral ischemia-reperfusion injury: mechanisms and therapeutic implications

**DOI:** 10.3389/fphar.2026.1744884

**Published:** 2026-04-14

**Authors:** Xue Fang, Yin Fu, Yidi Ma, Bojia Li, Zhe Zhang, Taoran Mo, Aidi Li, Yuntian Xie, Qiang Fu

**Affiliations:** 1 Heilongjiang University of Traditional Chinese Medicine, Harbin, China; 2 School of Basic Medical Sciences, Heilongjiang University of Traditional Chinese Medicine, Harbin, China; 3 Department of Nephrology, The First Affiliated Hospital of Heilongjiang University of Traditional Chinese Medicine, Harbin, China

**Keywords:** autophagy-dependent ferroptosis, cell death, cerebral ischemia-reperfusion injury, myocardial ischemia-reperfusion injury, PANoptosis

## Abstract

Ischemia-reperfusion injury (IRI) represents a critical pathological process contributing to secondary tissue damage in cardiovascular and cerebrovascular diseases. The complexity of the cell death network involved poses substantial challenges for therapeutic intervention. Among emerging forms of regulated cell death, autophagy-dependent ferroptosis and PANoptosis have attracted considerable attention. This review aims to elucidate the potential formation of a highly coordinated cell death network in myocardial and cerebral IRI through the convergence of these two pathways via shared key molecules. By examining their molecular underpinnings, we focus on core regulators such as NLRP3, STING, RIPK, GPX4, and NCOA4, which not only drive their respective pathways but may also facilitate PANoptosome assembly and integrate death signals, thereby mediating signal amplification and crosstalk. Despite inherent differences between cardiac and cerebral tissues, this network may exert synergistic effects during IRI progression by sharing upstream oxidative stress and inflammatory signals. Although current evidence is largely derived from *in vitro* models, the *in vivo* interactions and clinical translational potential of these mechanisms remain to be further validated. We propose that future therapeutic strategies may shift from targeting individual pathways to intervening at critical crosstalk nodes, offering a new direction for developing more effective protective strategies against IRI in the heart, brain, and beyond.

## 1 Introduction

**FIGURE 1:**
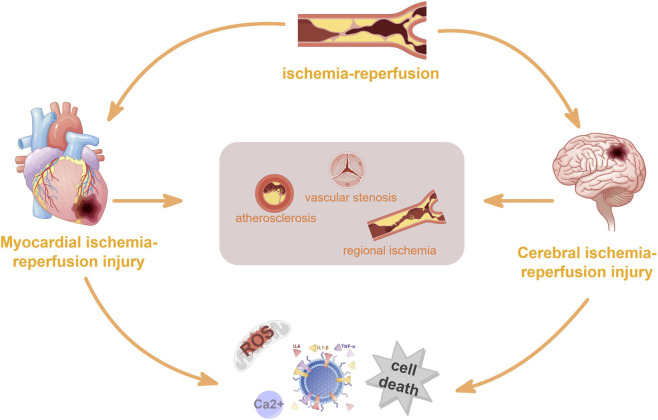
Schematic Overview of Ischemia-Reperfusion Injury in Cardiac and Cerebral Tissues: Schematic depicting the common pathological cascade of ischemia-reperfusion injury (IRI) in the heart and brain. Upstream triggers (vascular stenosis, atherosclerosis) induce regional ischemia in myocardial and cerebral tissues; subsequent reperfusion though necessary for tissue salvage, paradoxically exacerbates cellular damage and culminates in cell death, establishing IRI as a critical pathological basis for both organ injuries post-ischemia. (Source: Figdraw).

Ischemia-reperfusion injury (IRI) refers to the paradoxical exacerbation of tissue damage that occurs when blood flow is restored to previously ischemic or infarcted tissue. Rather than restoring structure and function, reperfusion triggers a complex cascade of pathophysiological events within the affected area. These events amplify pre-existing injury, impair organ function, cause irreversible secondary damage to cells, and ultimately expand the infarct area, leading to more extensive tissue damage and disease progression. IRI is thus recognized as a common pathological basis for multiple organ disorders (Wu et al., 2018). This phenomenon is most frequently observed in the heart and brain. The primary risk factors for IRI-related diseases are increasing in prevalence worldwide (Gu et al., 2024; Martin et al., 2025). Ischemic heart disease remains one of the leading causes of death globally, with both its incidence and mortality rising annually (Heusch, 2024). Although reperfusion strategies such as percutaneous coronary intervention, coronary artery bypass grafting, and pharmacological thrombolysis restore blood flow and oxygen supply to ischemic myocardium, they can ultimately trigger ventricular remodeling (Moon et al., 2020). Experimental studies have indicated that myocardial ischemia-reperfusion injury (MIRI) directly or indirectly accounts for up to 50% of the final infarct size. Therefore, further investigation into the molecular mechanisms underlying MIRI-induced myocardial injury is of critical importance (Cereda et al., 2026). Cerebrovascular disease is the second leading cause of death worldwide (Louca et al., 2026). In clinical practice, mechanical thrombectomy and intravenous administration of plasminogen activators are commonly employed to restore blood supply to ischemic brain tissue as rapidly as possible. However, the secondary injury that compromises neurological function after reperfusion, termed CIRI, remains a major challenge (Wu et al., 2020). Both CIRI and MIRI share a common primary etiology: reduced regional blood flow due to vascular stenosis or atherosclerosis (Gunata and Parlakpinar, 2021). Accumulating clinical and experimental evidence suggests a close causal relationship between brain injury and cardiac dysfunction (Li et al., 2024; Zeng et al., 2024). Moreover, studies have demonstrated that the pathophysiological and phenotypic mechanisms underlying cell death and inflammation in cerebral ischemic stroke (IS) resemble those observed in certain cardiac conditions (He and Zhang, 2020; Yan et al., 2022). Both cerebral and myocardial IRI involve core mechanisms such as oxidative stress, calcium overload, inflammatory mediators, and cell death (Bompotis et al., 2016; Jiang et al., 2020; Murphy and Steenbergen, 2008; Kong et al., 2022). These factors intersect and collectively trigger regulated cell death, forming key nodes in disease initiation and progression. The common pathological basis of cardiac and cerebral ischemia-reperfusion injury is illustrated in Figure 1. Recent studies have uncovered interactions between autophagy-dependent ferroptosis and PANoptosis in the context of IRI. Elucidating the crosstalk among these pathways and the key molecules involved may deepen our understanding of IRI pathophysiology and provide a theoretical basis for developing novel therapeutic strategies.

Cell death is a fundamental biological process in multicellular organisms. Based on whether it is controlled and involves specific molecular mechanisms, currently known forms of cell death can be classified into three major categories: accidental cell death (ACD), regulated cell death (RCD), and programmed necrosis (Galluzzi et al., 2018). Among the emerging forms of RCD, autophagy-dependent ferroptosis and PANoptosis have attracted considerable attention due to their complex signaling pathways and multifactorial interactions. Ferroptosis was first characterized by the Stockwell Lab in 2012. It is defined as an iron-dependent form of regulated necrosis, driven by an imbalance in the expression and activity of redox-active enzymes. These enzymes either generate reactive oxygen species or eliminate free radicals and lipid peroxidation products, ultimately leading to membrane damage (Tang D. et al., 2021; Ru et al., 2024). The core features of ferroptosis include depletion of intracellular glutathione (GSH) and inactivation of glutathione peroxidase 4 (GPX4). Under these conditions, excessive lipid peroxides cannot be cleared via GPX4-catalyzed reduction, thereby triggering ferroptotic cell death (Yu et al., 2021). Autophagy is a lysosomal degradation pathway that enables the recycling of cellular components through autophagosome formation and subsequent hydrolysis. It is regulated and executed by a set of conserved proteins known as autophagy-related proteins (ATGs), which form distinct complexes to shape membrane dynamics and facilitate substrate encapsulation and degradation. Autophagy plays a critical role in maintaining cellular homeostasis, particularly under nutrient-deprived conditions (Klionsky and Emr, 2000). Recent studies have revealed crosstalk between autophagy and ferroptosis. A distinct form of ferroptosis actively driven by selective autophagy has been termed autophagy-dependent ferroptosis (Chen X. et al., 2024). The mechanisms underlying autophagy-dependent ferroptosis primarily involve several forms of selective autophagy, including ferritinophagy, lipophagy, clockophagy, and other selective autophagic processes. Ferritinophagy, mediated by NCOA4, degrades ferritin to release labile iron; lipophagy degrades lipid droplets to release polyunsaturated fatty acids (substrates for lipid peroxidation); and clockophagy degrades key anti-ferroptotic proteins such as GPX4 and FSP1. Excessive activation of these selective autophagy pathways can provide pro-death substrates and initiate ferroptosis (Liu et al., 2023). In 2019, Kanneganti and colleagues first described PANoptosis (Tang et al., 2019). This process involves Z-DNA binding protein 1 (ZBP1) acting as a sensor for influenza A virus (IAV) proteins NP and PB1, thereby triggering both cell death and inflammatory responses. PANoptosis is defined as an inflammatory programmed cell death phenotype that integrates key features of pyroptosis, apoptosis, and necroptosis, but cannot be fully explained by any single one of these pathways (Pandian and Kanneganti, 2022). The PANoptosome, a multi-protein complex assembled from specific sensors, adaptors, and effectors, drives PANoptosis, and its assembly is a prerequisite for initiating this cell death modality. As emerging forms of regulated cell death (RCD), autophagy-dependent ferroptosis and PANoptosis involve complex signaling networks and interactions. This review aims to explore the molecular regulatory mechanisms underlying these two processes and analyze their potential roles in the pathogenesis of MIRI and CIRI, aiming to identify novel therapeutic targets for ischemic diseases (Galluzzi et al., 2018).

## 2 Molecular mechanisms: core regulatory networks of autophagy-dependent ferroptosis and PANoptosis

### 2.1 Dual regulatory mechanisms of autophagy-dependent ferroptosis

**FIGURE 2:**
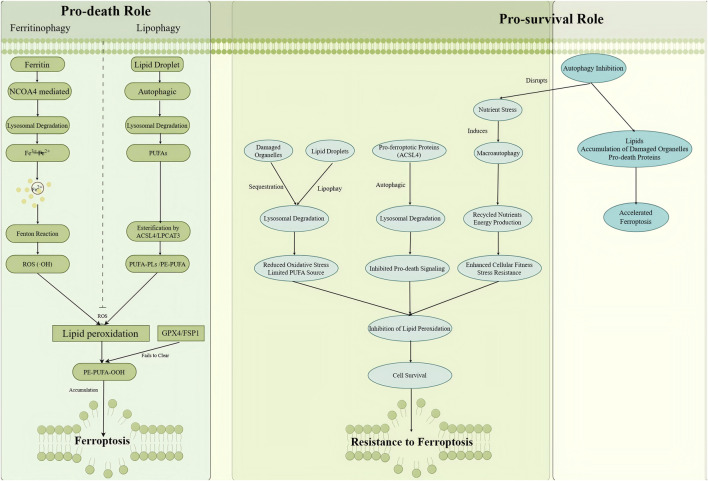
Dual Roles of Autophagy in Ferroptosis Regulation: Schematic depicting the context-dependent functions of autophagy in ferroptosis. Autophagy promotes ferroptosis through selective pathways: NCOA4-mediated ferritinophagy releases labile iron (Fe^2+^), driving the Fenton reaction and lipid peroxidation; lipophagy supplies polyunsaturated fatty acids (PUFAs) as lipid peroxidation substrates; and degradation of anti-ferroptotic proteins (e.g., GPX4) cripples cellular defenses. Conversely, autophagy exerts pro-survival functions by clearing damaged organelles to reduce oxidative stress, recycling nutrients for energy homeostasis, and degrading pro-ferroptotic proteins (e.g., ACSL4). The balance between these opposing forces determines ferroptosis fate. ACSL4, acyl-CoA synthetase long-chain family member 4; GPX4, glutathione peroxidase 4; NCOA4, nuclear receptor coactivator 4; PUFA, polyunsaturated fatty acid; ROS, reactive oxygen species. (Source: Figdraw).

In this review, we focus specifically on the pro-death aspect of autophagy-dependent ferroptosis, the mechanisms by which selective autophagy actively promotes ferroptosis through the release of labile iron, provision of lipid substrates, or degradation of anti-ferroptotic proteins. The pro-survival functions of autophagy, while acknowledged, are not the primary focus of this discussion. Autophagy-dependent ferroptosis refers specifically to the process in which selective autophagy participates in the initiation and execution of ferroptosis by degrading specific anti-ferroptotic molecules or providing pro-ferroptotic substrates. Depending on cell type, stress conditions, and signaling pathways, specific types of selective autophagy can either promote or inhibit ferroptosis by supplying critical “execution substrates.” The currently known molecular mechanisms underlying autophagy-dependent ferroptosis can be classified into three main categories, with ferritinophagy being the most well-characterized. In this process, the selective autophagy receptor NCOA4 recognizes ferritin and transports it to autolysosomes for degradation, releasing substantial amounts of labile intracellular iron. This iron subsequently drives hydroxyl radical production via the Fenton reaction, initiating lipid peroxidation and culminating in ferroptosis. Lipophagy represents another mechanism wherein autophagy regulates lipid metabolism-related proteins, degrades lipid droplets, and releases stored polyunsaturated fatty acids (PUFAs). These PUFAs are incorporated into membrane phospholipids through enzymatic catalysis by ACSL4 and LPCAT3, thereby promoting lipid peroxide accumulation. Additionally, autophagy can selectively degrade ferroptosis suppressor proteins, such as glutathione peroxidase 4 (GPX4) and ferroptosis suppress or protein 1 (FSP1) or specific organelles, thereby diminishing the cellular capacity to clear lipid peroxides and enhancing sensitivity to ferroptosis (Wang et al., 2025). At the molecular level, loss of Hippo pathway effectors YAP/TAZ has been indicated to activate autophagy and promote ferroptosis through NCOA4-dependent ferritinophagy. Autophagy exerts dual functions in cellular homeostasis. Beyond the ferroptosis-promoting mechanisms described above, selective autophagy can also remove damaged organelles to reduce oxidative stress and prevent lipid peroxidation, or selectively degrade pro-ferroptotic proteins to inhibit ferroptosis. Furthermore, autophagy maintains metabolic homeostasis by recycling intracellular components, preserving energy and metabolic balance, and eliminating potential ferroptosis triggers processes that enhance cellular resistance to ferroptosis and exert protective effects (Chu et al., 2023; Zhu Y. et al., 2025). Such protective functions do not contradict the concept of autophagy-dependent ferroptosis but reflect the functional diversity of autophagy. This review focuses specifically on the ferroptosis-promoting aspect of autophagy. In this context, autophagy-dependent ferroptosis refers to autophagy facilitating and accelerating ferroptosis through the release of labile iron, provision of lipid substrates, or degradation of ferroptosis-protective proteins. Consistent with this framework, pharmacological inhibition of autophagy (e.g., chloroquine, CQ) or knockdown of core autophagy genes (e.g., ATG5, ATG7) has been indicated to significantly suppress ferroptosis (Li et al., 2021). The dual regulatory roles of autophagy in ferroptosis are depicted in Figure 2.

### 2.2 PANoptosome assembly and its regulatory mechanisms

It is important to distinguish between PANoptosis as the cell death phenotype and the PANoptosome as the molecular platform that executes it. PANoptosome assembly is a prerequisite for PANoptosis, but the presence of PANoptosome components does not necessarily indicate active PANoptosis, as these molecules may also participate in other cellular processes. PANoptosis is a programmed cell death phenotype regulated by the PANoptosome complex, and it exhibits concurrent features of apoptosis, necroptosis, and pyroptosis. The PANoptosome is a multimeric complex that integrates components from multiple cell death pathways, primarily sensor proteins (ZBP1, AIM2, NLRP3), adaptor proteins (ASC and FAS-associated death domain protein FADD), and catalytic effectors (RIPK1/3, caspase-1/8) (Qi et al., 2023; Sundaram et al., 2023). Its molecular mechanisms involve multiple regulatory levels. PANoptosome assembly constitutes the core upstream event triggering PANoptosis. Studies in infectious disease models have indicated that upon sensing pathogenic infection or endogenous danger signals, specific sensor proteins (e.g., ZBP1, AIM2, RIPK1, and NLRP12) become activated and serve as scaffolds to recruit additional molecules, thereby initiating PANoptosome assembly. The assembled PANoptosome subsequently activates downstream effectors (caspase activation, GSDMD cleavage, and MLKL phosphorylation), ultimately leading to membrane pore formation and PANoptosis execution (Zheng and Kanneganti, 2020; Place et al., 2020). Whether similar mechanisms are activated in ischemia-reperfusion injury remains under investigation. However, recent studies suggest that ZBP1 is highly expressed in MIRI and may facilitate PANoptosome assembly (Zhang Y. et al., 2025; Zhu L. et al., 2025).

As a newly identified pro-inflammatory programmed cell death pathway, PANoptosis is a potential therapeutic target for ischemic diseases. Z-DNA binding protein 1 (ZBP1) functions as an upstream molecule that transmits cell death signals and senses aberrant nucleic acid production. Additionally, ZBP1 serves as a scaffold protein that integrates cell death signals and facilitates PANoptosome assembly (Kuriakose and Kanneganti, 2017; Hao et al., 2022). Through assembly of the ZBP1-dependent PANoptosome, ZBP1 activates NLRP3, ASC, and caspases 1/6 to facilitate Gasdermin D (GSDMD)-dependent pyroptosis (Zheng and Kanneganti, 2020); activates FADD and caspase-8 to induce caspase-3/7-mediated apoptosis; and activates RIPK1 and RIPK3 to promote MLKL phosphorylation-mediated necroptosis (Briard et al., 2021). Through these three pathways, ZBP1-driven PANoptosome assembly contributes to PANoptosis induction (Qi et al., 2023). Absent in melanoma 2 (AIM2) responds to Francisella and herpes simplex virus 1 (HSV-1) infection by forming a PANoptosome. This process involves cooperative assembly between the AIM2 inflammasome pyrin domain and ZBP1, thereby initiating PANoptosis (Place et al., 2020; Man et al., 2015). AIM2 recruits ASC and caspase-1 to form an inflammasome that facilitates pyroptosis via GSDMD cleavage, activates caspase-3/7 to mediate apoptosis, and induces MLKL-mediated necroptosis. Coordinated activation of these pathways characterizes PANoptosis triggered by AIM2-driven PANoptosome assembly (Lee et al., 2021). Receptor-interacting serine/threonine-protein kinase 1 (RIPK1) serves as a critical component of the PANoptosome that integrates multiple death signals (Degterev et al., 2008). RIPK1 activates the NLRP3 inflammasome, leading to caspase-1-mediated GSDMD cleavage and subsequent membrane pore formation to induce pyroptosis. Concurrently, RIPK1 recruits FADD through its kinase activity to activate caspase-8, which initiates caspase-3/7 cleavage and triggers apoptosis (Bock and Tait, 2020). Additionally, RIPK1 interacts with RIPK3 via its RHIM domain to form the necrosome, resulting in MLKL phosphorylation and necroptosis induction (Ying et al., 2021; Grootjans et al., 2017). Through simultaneous activation of these three pathways, RIPK1 facilitates PANoptosome functional execution and contributes to PANoptosis induction (Qi et al., 2023). NOD-like receptor family pyrin domain containing 12 (NLRP12) forms an inflammasome with ASC and caspase-1 to drive pyroptosis (Shami et al., 2001). In response to heme and pathogen-associated molecular patterns (PAMPs), NLRP12 additionally serves as a scaffold that recruits ASC, caspase-8, and RIPK3 to assemble the PANoptosome complex, thereby initiating PANoptosis (Sundaram et al., 2023; Vladimer et al., 2012). Interferon regulatory factor 1 (IRF1) upregulates ZBP1 expression during influenza A virus (IAV) infection, promoting ZBP1-PANoptosome formation and subsequent PANoptosis. In the AIM2 inflammasome, IRF1 functions as a regulatory molecule that participates in PANoptosis initiation together with caspase-8 and RIPK3 as components of the PANoptosome. Moreover, IRF1 acts as an upstream regulator of the NLRP12-PANoptosome, responding to NLRC4 inflammasome and caspase-11-mediated pathogen infection signals (Sundaram et al., 2023; Kuriakose et al., 1950). Caspases represent a distinct family of cysteinyl aspartate-specific proteinases that drive innate immune-mediated inflammatory programmed cell death pathways. These processes are regulated by PANoptosome complexes and involve coordinated activation of caspases and receptor-interacting protein kinases (RIPKs) (Sharma et al., 2023; Nguyen et al., 2022).

## 3 Cross-talk of key signaling molecules

### 3.1 ROS as a hub: positive feedback loops

Reactive oxygen species (ROS) not only exacerbate cellular damage but also serve as critical signaling molecules connecting distinct cell death programs. During ischemia-reperfusion injury, autophagy activation promotes oxidative stress, leading to a significant elevation in ROS levels. Within the framework of autophagy-dependent ferroptosis, the autophagy adaptor protein NCOA4 mediates ferritinophagy by transporting ferritin to lysosomes for degradation. This process releases substantial amounts of labile iron ions, which catalyze the Fenton reaction to generate toxic hydroxyl radicals (•OH). These radicals oxidize polyunsaturated fatty acids (PUFAs), initiating a chain reaction of lipid peroxidation (Huang et al., 2023). Concurrently, lipophagy degrades lipid droplets to release abundant PUFAs, which are incorporated into membrane phospholipids via ACSL4 and LPCAT3, thereby providing substrates for lipid peroxidation. Additionally, autophagy can degrade GPX4 or glutathione synthesis-related proteins, reducing the cellular capacity to clear lipid peroxides (Chen X. et al., 2024).

Collectively, these mechanisms constitute the core events underlying autophagy-dependent ferroptosis. Elevated ROS levels further impair mitochondria, leading to mitochondrial dysfunction and the subsequent generation of additional ROS. Meanwhile, ROS can directly oxidize autophagy-related proteins, thereby further activating autophagy. This forms a sustained positive feedback loop that contributes to lipid peroxide accumulation and plasma membrane rupture, ultimately leading to ferroptosis (Yu et al., 2021). *In vitro* models have demonstrated that ROS and lipid peroxides accumulated during ferroptosis can activate the NLRP3 inflammasome, leading to GSDMD cleavage and pyroptosis induction, thereby linking ferroptosis with PANoptosis (Du et al., 2025). Emerging evidence suggests that ROS and lipid peroxidation products may serve as signaling bridges connecting autophagy-dependent ferroptosis with downstream inflammatory cell death. In cerebral ischemia-reperfusion injury models, autophagy inhibition has been indicated to significantly reduce ROS accumulation during ferroptosis and synergistically suppress NLRP3/caspase-1/GSDMD-mediated cell death (Du et al., 2025). Based on these findings, we speculate that similar mechanisms may operate in myocardial ischemia injury, providing potential insights for therapeutic strategies targeting MIRI. The differential roles of ROS in MIRI versus CIRI are summarized in Table 1, highlighting tissue-specific activation patterns of downstream inflammasomes.

**TABLE 1:** Comparison of autophagy-dependent ferroptosis and PANoptosis in myocardial versus cerebral ischemia-reperfusion injury.

Feature	Myocardial ischemia-reperfusion injury (MIRI)	Cerebral ischemia-reperfusion injury (CIRI)
Clinical Manifestations	Predominantly circulatory symptoms: chest pain, chest tightness, hypotension, arrhythmias	Predominantly neurological symptoms: hemiplegia, speech disturbances, altered consciousness, seizures
Injury Site and Cell Types	Primary injury to cardiomyocytes, directly leading to decreased cardiac function (e.g., arrhythmias, heart failure) (Heusch, 2024; Sun et al., 2023; Fuhrmann et al., 2020; Zhao et al., 2024; Zhang et al., 2024; Ito et al., 2021)	Injury to neurons, astrocytes, and microglia, resulting in neurological deficits (e.g., limb paralysis, cognitive impairment, coma) (Louca et al., 2026; Liu et al., 2022; Naito et al., 2020)
Reperfusion Strategy Differences	Emphasis on “rapid recanalization” (e.g., thrombolysis, PCI); concurrent prevention of reperfusion arrhythmias required (Moon et al., 2020)	Following recanalization, focus on blood-brain barrier protection and prevention of cerebral edema (e.g., mannitol); avoidance of intracranial hypertension (Wu et al., 2020)
Structural Features and Drug Delivery	Absence of blood-brain barrier; drugs readily access the injury site (Moon et al., 2020); Cardiomyocytes rely on aerobic metabolism and are more sensitive to ischemia (Heusch, 2024)	Presence of blood-brain barrier; high drug permeability required (Wu et al., 2020; Luedde et al., 2014); Neurons are non-regenerative, making complete recovery difficult following injury (Liu et al., 2022)
Upstream Trigger Characteristics	Oxygen/nutrient deprivation; Calcium overload (Bompotis et al., 2016; Jiang et al., 2020); Mitochondrial dysfunction (Murphy and Steenbergen, 2008; Kong et al., 2022)	Oxygen/glucose deprivation (Wu et al., 2020); Glutamate excitotoxicity (Malireddi et al., 2019); Neuroinflammation (Chen et al., 2021)
Predominant Cell Death Modes	Autophagy-dependent ferroptosis serves as the initial driver in early ischemia (Li et al., 2023; Wu et al., 2024b); PANoptosis acts as an inflammatory amplifier (Zhu et al., 2025b; Kuriakose and Kanneganti, 2017; Zheng et al., 2022)	Multiple cell death modes coexist; PANoptosis is particularly prominent in glial cells (Yan et al., 2022; Liu et al., 2022); Ferroptosis often occurs as a secondary or parallel event (Liao et al., 2026)
Inflammatory Amplification Hub	NLRP3 inflammasome; robustly activated by ROS and lipid peroxides (Liu et al., 2021; Motwani et al., 2019; Tsuchiya et al., 2019)	NLRP3 synergistically activated with multiple inflammasomes, including NLRC4 (Luedde et al., 2014; Poh et al., 2019; Yang et al., 2017)
Key Regulatory Nodes - Iron Metabolism	NCOA4-mediated ferritinophagy constitutes critical iron supply mechanism (Li et al., 2023; Wu et al., 2024b); ENPP2-SIRT1/PGC-1α/NRF1 pathway provides endogenous protection (Chapman et al., 2025)	NCOA4 participates in iron metabolism regulation; the cGAS-STING pathway functions as an upstream activating signal (Li et al., 2023; Fan et al., 2023; Liao et al., 2026)
Key Regulatory Nodes - Death Integration	ZBP1/RIPK1 mediates PANoptosome assembly (Zhu et al., 2025b; Kuriakose and Kanneganti, 2017); p53 integrates multiple death signals upstream (Tang et al., 2021b; Qu et al., 2024; She et al., 2023)	TAK1 is critical switch for neuronal death (Malireddi et al., 2019; Chueh et al., 2020); RIPK3 exhibits dual death execution and inflammatory signaling functions (Karki et al., 2021b; Johnson et al., 2024; Hu et al., 2022); USP13 influences death network through autophagy regulation (Johnson et al., 2024)
Shared Hub - STING	STING upregulates ZBP1 through type I interferon activation, triggering PANoptosis (Gong et al., 2020; Oduro et al., 2022; Sarhan et al., 2019)	cGAS-STING pathway activates NCOA4-mediated ferritinophagy, promoting ferroptosis (Luedde et al., 2014; Li et al., 2023; Fan et al., 2023)
Shared Hub - RIPK Family	RIPK1/RIPK3/MLKL pathway activation mediates necroptosis (Newton et al., 2016; Hu et al., 2019); Targeting this pathway improves cardiac function (Hu et al., 2019; DeRoo et al., 2020; Liao et al., 2020; W et al., 2023)	RIPK3 mediates necroptosis and participates in inflammatory signal transduction (Karki et al., 2021b; Hu et al., 2022); Pathway inhibition reduces cerebral infarct size (Karki et al., 2021b; Johnson et al., 2024)
Shared Hub - GPX4	GPX4 is critical ferroptosis suppressor protein; Autophagic degradation of GPX4 promotes ferroptosis (Wang et al., 2025); Targeting GPX4/RIPK axis confers synergistic cardioprotection (W et al., 2023)	Functional crosstalk exists between GPX4 antioxidant function and RIPK pathways, jointly regulating cell death (Chen et al., 2021)
Therapeutic Strategies - Direct Targets	NLRP3 inhibitors attenuate myocardial injury in mouse MIRI models (Tsuchiya et al., 2019); RIPK1 inhibitor (Nec-1) improves cardiac function in rat MIRI models (Hu et al., 2019); Ferroptosis inhibitors (Fer-1, DFO) protect myocardium in rat MIRI models (W et al., 2023) and in mouse CIRI models (Li et al., 2023)	RIPK3 inhibitors reduce ischemic brain injury in mouse CIRI models (Karki et al., 2021b; Johnson et al., 2024); TAK1 inhibitors (Malireddi et al., 2019) and USP13-targeted interventions (Johnson et al., 2024) show therapeutic potential *in vitro* and *in vivo* models
Therapeutic Strategies - Multi-Target Agents	Salvia miltiorrhiza decoction targets miR-93-5p to inhibit TXNIP/NLRP3/caspase-1 pathway in mouse MIRI models (Chen et al., 2024b; Tsuchiya et al., 2019); Xianling Gubao capsule inhibits NLRP3/caspase-3/RIP1-mediated PANoptosis in mouse MIRI models (Zheng et al., 2022)	Ginsenoside Rb3 modulates both inflammation and ferroptosis via NLRP3/NF-κB/GPX4 pathway in rat CIRI models (Chen et al., 2021); Edaravone dexborneol inhibits the cGAS-STING pathway to attenuate CIRI in rat CIRI models and *in vitro* microglia models (Luedde et al., 2014)
Therapeutic Strategies - Combined Interventions	Combined inhibition of apoptosis (Z-vad) and ferroptosis (Fer-1) demonstrates cardioprotection in rat MIRI models (W et al., 2023)	Targeting GATA6/miR-193b/ATG7 axis simultaneously inhibits neuronal autophagy and ferroptosis in mouse CIRI models and *in vitro* neuronal models (Liao et al., 2026)

### 3.2 Iron metabolism: coordinated regulation at key nodes

Dysregulation of iron metabolism is a critical link connecting different cell death modalities. Studies have indicated that the STAT3 signaling pathway is persistently activated in high-fat diet-induced cardiac injury, where it transcriptionally upregulates nuclear receptor coactivator 4 (NCOA4) expression. NCOA4 subsequently recognizes and directs ferritinophagy, thereby contributing to autophagy-dependent ferroptosis. Concurrently, STAT3 functions as a transcription factor involved in NLRP3 inflammasome activation, which facilitates IL-1β and IL-18 release and pyroptosis induction (Zhu et al., 2023). These findings suggest that upon sensing metabolic stress, STAT3 may serve as an upstream regulator simultaneously controlling both the NCOA4-ferroptosis axis and the NLRP3-pyroptosis axis. Based on this dual regulatory role of STAT3 in two distinct cell death pathways, we hypothesize that STAT3 may participate in integrating ferroptotic and inflammatory death signals, thereby potentially contributing to PANoptosome assembly and PANoptosis regulation. However, this hypothesis requires direct validation in ischemia-reperfusion injury models. The tissue-specific regulatory patterns of iron metabolism, including the role of NCOA4, are compared in Table 1.

### 3.3 Lipid metabolism: shared substrates

**FIGURE 3:**
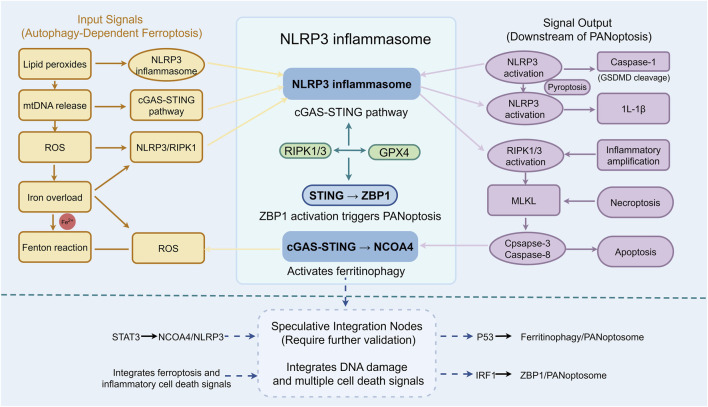
Key Molecular Intersection Nodes Orchestrating the Crosstalk Between Autophagy-Dependent Ferroptosis and PANoptosis in Ischemia-Reperfusion Injury (IRI): Focused schematic highlighting the core signaling hubs that integrate autophagy-dependent ferroptosis and PANoptosis. The NLRP3 inflammasome and RIPK1/RIPK3 complex serve as central platforms receiving inputs from ferroptosis-derived signals-including lipid peroxides, mitochondrial DNA (mtDNA) via the cGAS-STING pathway, and reactive oxygen species (ROS), which activate downstream effectors. Key outputs include caspase-1-mediated pyroptosis, MLKL-mediated necroptosis, and caspase-8/caspase-3-mediated apoptosis. Bidirectional crosstalk between RIPK1/3 and GPX4 is depicted, where targeting this axis provides synergistic cardioprotection. Speculative integrative nodes (STAT3, p53, IRF1) requiring further validation are outlined in the dashed box, representing potential links among ferroptosis, inflammation, and PANoptosome assembly. Solid arrows indicate mechanisms supported by direct experimental evidence; dashed arrows represent speculative or indirect regulatory relationships; bidirectional arrows denote functional crosstalk between pathways. (Source: Figdraw).

Lipid metabolic reprogramming serves as a source of common molecular substrates for the cross-talk between different cell death modalities. Acyl-CoA synthetase long-chain family member 4 (ACSL4) is critical for ferroptosis, as it catalyzes the activation of unsaturated fatty acids and facilitates the synthesis of membrane phospholipids susceptible to lipid peroxidation. Notably, lipid peroxidation products also act as inflammatory signals that can further accelerate PANoptosis. Recent studies have indicated that icariin significantly attenuates lipid peroxidation and prevents glutathione peroxidase 4 (GPX4) depletion during ferroptosis. The underlying mechanism may involve autophagy regulation through inhibition of the IRE1/JNK pathway, thereby modulating multiple stress response-mediated signals, including apoptosis and inflammation. These findings suggest that icariin may exert protective effects against ischemia-reperfusion injury by intervening in the cross-talk between autophagy-dependent ferroptosis and PANoptosis (Rochette et al., 2022). The key molecular intersection nodes orchestrating the crosstalk between autophagy-dependent ferroptosis and PANoptosis are shown in Figure 3.

## 4 Cross-regulatory network: interplay between autophagy-dependent ferroptosis and PANoptosis

### 4.1 Autophagy-dependent ferroptosis and pyroptosis

During autophagy-dependent ferroptosis, selective autophagic processes such as NCOA4-mediated ferritinophagy contribute to increased reactive oxygen species (ROS) generation. Under oxidative stress conditions, this can activate the NLRP3 inflammasome, thereby initiating pyroptotic programs. NLRP3 activation subsequently induces caspase-1, which cleaves gasdermin D (GSDMD) to form the GSDMD-N fragment. This disrupts cellular ionic homeostasis and creates a more favorable environment for lipid peroxidation. Concurrently, caspase-1 promotes the release of pro-inflammatory cytokines IL-1β and IL-18, further exacerbating oxidative stress and potentiating ferroptosis. Studies have revealed a positive correlation between the expression of pyroptosis-related proteins and autophagy markers, suggesting that pyroptosis activation may depend on autophagic processes and potentially further drive ferroptotic progression (Liu et al., 2021; Lu et al., 2021). p53 has been implicated in both mediating autophagy-dependent ferroptosis (Wang et al., 2023; Liu et al., 2025) and participating in PANoptosis through regulation of inflammatory responses. Similarly, HMGB1 is thought to contribute to the interplay between these two death modalities by shaping the inflammatory microenvironment (Wen et al., 2019; Wei et al., 2024). These findings suggest that autophagy-dependent ferroptosis and pyroptosis may exhibit synergistic effects, wherein activation of one pathway potentiates the other, thereby exacerbating tissue injury.

### 4.2 Autophagy-dependent ferroptosis and apoptosis

Ischemia-reperfusion, as an upstream stress stimulus, can simultaneously activate both autophagy-dependent ferroptosis and apoptosis, with significant interplay between these two pathways. Studies have indicated that activated caspase-3 cleaves gasdermin E (GSDME), releasing the GSDME-N fragment that targets the plasma membrane to induce pyroptosis. Conversely, GSDME-N can permeabilize mitochondria, which contributes to caspase-3 activation during cell death and inflammasome activation. This subsequently downregulates SLC7A11, a subunit of system xc^−^, reducing cystine uptake and suppressing GPX protein expression, thereby contributing to autophagy-dependent ferroptosis (Yin et al., 2022; Yin et al., 2024; Deng et al., 2025). Conversely, the abundant lipid peroxides released during lipophagy can also damage mitochondria, leading to membrane potential collapse, GSDME activation, and subsequent apoptosis promotion. This bidirectional interplay reflects the synergistic amplification between autophagy-dependent ferroptosis and apoptosis. Under certain conditions, autophagy-dependent ferroptosis and apoptosis can synergistically accelerate cell death, while cells may also switch between death modalities depending on stress context and duration. The interdependence of ferroptosis and apoptosis was highlighted by Qiu et al. (2025), who found that impaired GPX4 activity enables the simultaneous manifestation of both death programs, challenging the view of them as entirely distinct pathways. Furthermore, BH3-mimetics targeting the BCL-2 family can both synergistically enhance overall cell death and convert ferroptosis to apoptosis. When one apoptotic pathway is inhibited, cells may be redirected toward ferroptotic pathways (He et al., 2024).

### 4.3 Autophagy-dependent ferroptosis and necroptosis

**FIGURE 4:**
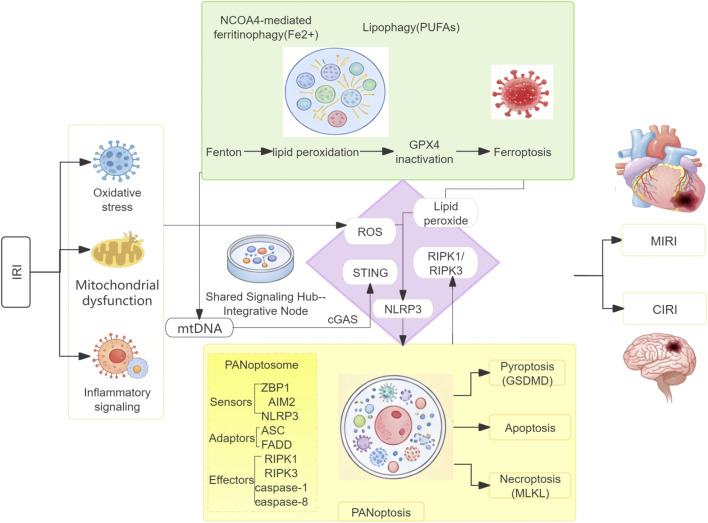
Synergistic Interaction Network of Autophagy-Dependent Ferroptosis and PANoptosis in Ischemia-Reperfusion Injury (IRI): Schematic overview of the proposed cooperative cell death network in myocardial and cerebral IRI. Upstream IRI stresses (oxidative stress, mitochondrial dysfunction, inflammation) initiate autophagy-dependent ferroptosis-driven by NCOA4-mediated ferritinophagy, lipophagy, and GPX4 inactivation-and PANoptosome assembly, which integrates pyroptotic, apoptotic, and necroptotic machinery. Key crosstalk is mediated by shared signals (ROS, lipid peroxides) and molecular hubs (NLRP3, RIPK kinases), forming a pro-death feedback loop. Tissue-specific outcomes in the heart and brain are highlighted on the right.

During autophagy-dependent ferroptosis, reactive oxygen species (ROS) generated through mechanisms such as ferritinophagy and lipophagy can activate RIPK1 and RIPK3, promoting necrosome formation and subsequently contributing to necroptosis. Concurrently, inflammatory cytokines such as TNF-α, which serve as inducers of necroptosis, can bind to TNFR1 on the cell membrane and activate downstream NF-κB signaling, thereby downregulating GPX4 transcription. Alternatively, TNF-α can activate the canonical RIPK1/RIPK3/MLKL pathway, leading to MLKL phosphorylation, activation, plasma membrane rupture, and glutamate release, which subsequently inhibits system xc^−^ function. Both mechanisms contribute to glutathione (GSH) depletion, reduced GPX4 synthesis, and diminished GPX4 activity, ultimately contributing to lipid peroxide accumulation and ferroptosis (Yang et al., 2022; Xie et al., 2025). A mechanistic link between FOXC1 and autophagy-dependent ferroptosis in MIRI was established by Chen et al. They demonstrated that FOXC1, activated under ischemic stress, transcriptionally upregulates ELAVL1. This stabilizes Beclin-1 mRNA, thereby promoting autophagy-dependent ferroptosis and exacerbating myocardial injury. Inhibition of this pathway significantly attenuated both ferroptosis and I/R injury, supporting the positive regulatory relationship between autophagy and ferroptosis (Chen et al., 2021). Autophagy-dependent ferroptosis and necroptosis share upstream activating signals, enabling them to mutually potentiate and accelerate the cell death process. Additionally, these pathways may exhibit competitive dynamics, wherein cells can switch from one death modality to another during the cross-talk between these two pathways. A panoramic view of the synergistic interaction network between autophagy-dependent ferroptosis and PANoptosis is presented in Figure 4.

## 5 Role of core molecules connecting ferroptosis and PANoptosis in myocardial and cerebral ischemia-reperfusion injury

Building upon the molecular mechanisms discussed above, this section focuses on the specific roles of key nodal molecules-including NLRP3, STING, RIPK family members, GPX4, and NCOA4-in myocardial and cerebral ischemia-reperfusion injury (IRI). Cardiac and cerebral tissues exhibit substantial differences in structure, cellular composition, and responses to ischemic insult, which influence the activation patterns of the cell death network, as summarized in Table 1.

### 5.1 Shared inflammatory amplifiers: NLRP3 inflammasome and STING

In this review, we focus specifically on the pro-death aspect of autophagy-dependent ferroptosis, the mechanisms by which selective autophagy actively promotes ferroptosis through the release of labile iron, provision of lipid substrates, or degradation of anti-ferroptotic proteins. The pro-survival functions of autophagy, while acknowledged, are not the primary focus of this discussion. Following initiation of autophagy-dependent ferroptosis, extensive lipid peroxidation generates abundant oxidized phospholipids, which contribute to mitochondrial dysfunction. Subsequent disruption of membrane integrity contributes to mitochondrial DNA (mtDNA) release into the cytoplasm, a critical event in both myocardial and cerebral IRI stress. Cytosolic mtDNA is recognized by cGAS, which synthesizes the second messenger cGAMP and can activate the STING pathway. Concurrently, potassium efflux promotes NLRP3 inflammasome assembly and activation (Zierhut and Funabiki, 2020; Motwani et al., 2019). Notably, as shown in Table 1, NLRP3 functions as an inflammatory amplification hub in both myocardial and cerebral IRI; however, its activation patterns differ between these tissues. In myocardial IRI, NLRP3 is predominantly activated by robust ROS and lipid peroxide signals, whereas in cerebral IRI, it is activated synergistically with multiple inflammasomes, including NLRC4. STING and NLRP3 can act synergistically to establish a pro-inflammatory microenvironment. Activated NLRP3 contributes to caspase-1-mediated release of IL-1β and IL-18, while STING pathway activation induces interferon-stimulated gene expression. Together, these signals shape an inflammatory milieu that potentiates cell death signaling. Caspase-1, as a core component of the PANoptosome, exhibits functional plasticity: when GSDMD-mediated pyroptosis execution is restricted, caspase-1 can directly switch death pathways by cleaving molecules such as caspase-3, thereby driving apoptosis or PANoptosis. Whether similar mechanisms contribute to cell death switching in the context of ischemia-reperfusion injury remains to be validated. Previous studies have indicated that exosomes derived from bone marrow mesenchymal stem cells (BMSCs) can exert therapeutic effects against MIRI (Mao et al., 2019; Li et al., 2019).MiRNA sequencing of exosomes isolated from BMSCs treated with Salvia miltiorrhiza decoction (Gong et al., 2023) indicated that this treatment targets miR-93-5p to inhibit activation of the TXNIP/NLRP3/caspase-1 signaling pathway. This results in downregulation of key inflammatory and apoptotic markers, including IL-1β, IL-18, caspase-1, NLRP3, TXNIP, and GSDMD, thereby attenuating cardiomyocyte pyroptosis and conferring cardioprotection (Chen M. et al., 2024). Caspase-1 acts as an upstream signal within the PANoptosome and can substitute for other upstream signals to recruit and activate caspase-7. In the absence of GSDMD, caspase-1 can directly switch pathways by cleaving caspase-3 to drive apoptosis (Tsuchiya et al., 2019; Mahib et al., 2020). Similarly, Xianling Gubao (XLGB) capsule has been indicated to attenuate myocardial injury in isoproterenol (ISO) induced cardiomyopathy models by inhibiting the NLRP3/caspase-3/RIP1-mediated PANoptosis pathway (Wu X. et al., 2024). Through myocardial infarction (MI) mouse models and qPCR analysis, Zhang and colleagues identified ten ferroptosis and autophagy related genes including IL-6, PTGS2, and JUN-as potential therapeutic targets in MI progression. These genes are involved in the regulation of IL-17 signaling, JAK-STAT signaling, and MAPK cascades (Zheng et al., 2022). Furthermore, STING activation can initiate type I interferon (IFN) production, which upregulates ZBP1 expression and can trigger PANoptosis (Karki et al., 2021a). This pathway is aberrantly activated in cardiovascular diseases, including MIRI, and its inhibition can effectively alleviate tissue injury (Gong et al., 2020; Oduro et al., 2022). STING pathway activation can induce multiple cell death modalities, including pyroptosis and necroptosis, and the integration of these death pathways is characteristic of PANoptosis (Sarhan et al., 2019). In CIRI research, Zhang and colleagues utilized rat middle cerebral artery occlusion/reperfusion models and OGD/R microglia models to indicate that edaravone dexborneol attenuates CIRI by inhibiting the cGAS-STING pathway, promoting STX17 mediated autophagic lysosomal fusion, and suppressing NLRP3 inflammasome activation and pyroptosis-related gene expression (Zhang X. et al., 2025). These findings support the cGAS-STING-NLRP3 axis as a potential critical node connecting these pathways. Therefore, further investigation of the STING-NLRP3-PANoptosis axis regulated by autophagy-dependent ferroptosis may reveal novel therapeutic targets for MIRI intervention.

### 5.2 Execution hubs of programmed cell death: RIPK family and GPX4

The receptor-interacting protein kinase (RIPK) family and glutathione peroxidase 4 (GPX4) act as key execution hubs in programmed cell death. Receptor-interacting protein kinase 3 (RIPK3), a member of the RIPK family alongside RIPK1, acts as a critical effector within the PANoptosome complex. Under stimulation by signals such as tumor necrosis factor-alpha (TNF-α), RIPK3 becomes activated, interacts with ZBP1, and phosphorylates MLKL to mediate necroptosis-a core molecular event in PANoptosis execution (Luedde et al., 2014; Newton et al., 2016; Karki et al., 2021b). As summarized in Table 1, RIPK3 exhibits more complex roles in CIRI compared to myocardial IRI. In CIRI, RIPK3 acts not only as a death executor but also participates in inflammatory signal transduction, reflecting the unique regulatory patterns of the cell death network in cerebral IRI. In MIR, this pathway is aberrantly activated. Studies have indicated significantly elevated mRNA and phosphorylated protein levels of both RIPK1 and RIPK3 in MIRI. Necrostatin-1 (Nec-1), a specific RIPK1 inhibitor, prevents RIPK1-RIPK3 interaction by binding to RIPK1 and inhibiting its kinase activity. This intervention substantially reduces necrotic cell death and inflammatory cell infiltration in cardiac tissue, thereby improving cardiac ejection fraction (Zhang et al., 2019; Hu et al., 2019; DeRoo et al., 2020). Notably, RIPK1/RIPK3/MLKL axis-mediated necroptosis is also operative in CIRI. Liao and colleagues indicated that inhibition of this pathway effectively reduces cerebral infarct size and improves neurological function (Liao et al., 2020), suggesting that targeting the RIPK1/RIPK3/MLKL axis may represent a common therapeutic strategy for ischemic injury in vital organs, including the heart and brain. However, RIPK3 exhibits context-dependent functional specificity. Recent studies have indicated that RIPK3 in vascular endothelial cells of adult animals can exert protective effects by maintaining vascular barrier integrity and attenuating inflammation through suppression of IL-6 secretion and monocyte adhesion (Johnson et al., 2024), underscoring the need for cell type-specific considerations in therapeutic targeting. The regulatory role of RIPK pathways in suppressing cell death has also been indicated in other oxidative stress-mediated disease models. Aspartic acid has been indicated to enhance antioxidant capacity through GPX4 upregulation, indirectly modulating RIPK1/RIPK3/MLKL protein levels and ultimately attenuating both cell death and tissue injury (Hu et al., 2022). In animal models, Luo and colleagues indicated that dual targeting of the GPX4/RIPK axis confers superior cardioprotective effects compared to targeting either pathway alone (Luo et al., 2022). These findings suggest functional crosstalk between GPX4-mediated antioxidant capacity and RIPK-mediated death pathways. We propose that targeting GPX4 may represent a strategy to intercept RIPK1/RIPK3-mediated PANoptosis, thereby ameliorating cell damage and inflammatory responses-potentially opening new avenues for therapeutic intervention in ischemia-reperfusion injury.

### 5.3 NCOA4: a dedicated mediator of autophagy-dependent ferroptosis

NCOA4 acts as a core executioner of autophagy-dependent ferroptosis by serving as a selective autophagy receptor that mediates ferritinophagy-the selective degradation of ferritin-thereby contributing to ferroptotic cell death. As summarized in Table 1, NCOA4-mediated ferritinophagy exhibits tissue-specific regulatory patterns in ischemia-reperfusion injury. In MIRI, NCOA4-dependent ferritinophagy acts as the primary source of labile iron for Fenton chemistry, initiating lipid peroxidation. In CIRI, NCOA4 similarly participates in iron metabolism regulation, although its upstream activating signals (such as the cGAS-STING pathway) may display tissue-specific characteristics. The post-translational regulation of NCOA4 was elucidated by Wu et al. Who showed that ATM kinase directly phosphorylates and stabilizes NCOA4 in response to DNA damage, thereby facilitating ferritinophagy. This contributes to ferritinophagy, increases intracellular labile iron, initiates lipid peroxidation, and ultimately contributes to ferroptosis (W et al., 2023). Given that oxidative stress during ischemia-reperfusion injury induces substantial DNA damage, NCOA4 may represent a critical hub connecting early stress signals to subsequent ferroptosis execution in both cardiac and cerebral IRI. In MIRI models, icariin has been indicated to attenuate ferroptosis and cardiomyocyte injury by inhibiting the endoplasmic reticulum stress sensor IRE1 and its downstream JNK signaling pathway (Zhong et al., 2024). Studies have indicated that endoplasmic reticulum stress (ERS) and JNK signaling act as key upstream regulators of NCOA4 expression and stability, suggesting that the IRE1/JNK axis likely contributes to autophagy-dependent ferroptosis through NCOA4 modulation (Sun et al., 2023; Fuhrmann et al., 2020). Zhao and colleagues indicated that NCOA4-mediated ferritinophagy is activated in CIRI. Silencing NCOA4 effectively attenuated oxygen-glucose deprivation/reperfusion (OGD/R)-induced cellular injury, while inhibition of the cGAS-STING pathway significantly suppressed ferritinophagy, oxidative stress, and cell damage (Zhao et al., 2024). Concurrent studies have indicated that the iron chelator deferoxamine (DFO) inhibits the cGAS-STING pathway and effectively mitigates NCOA4-induced ferritinophagy, thereby alleviating CIRI (Zhao et al., 2024; Li et al., 2023). Fan and colleagues indicated that the GATA6/miR-193b axis attenuates CIRI by downregulating ATG7, thereby suppressing neuronal autophagy and ferroptosis (Fan et al., 2023). These findings support NCOA4 as a potential critical hub that links early stress signals to ferroptosis execution in CIRI. Notably, beyond pro-death mechanisms, recent studies have also indicated endogenous protective pathways. Liao and colleagues indicated that ENPP2 significantly attenuates mouse MIRI-induced ferroptotic injury through activation of the SIRT1/PGC-1α/NRF1 signaling axis (Liao et al., 2026). This finding suggests that NCOA4-mediated ferritinophagy in cardiomyocytes operates within a multi-layered regulatory network. Therefore, targeting NCOA4-a key molecule in autophagy-dependent ferroptosis-may enable specific blockade of upstream ferroptotic signals, achieving more precise therapeutic regulation in ischemia-reperfusion injury.

## 6 Tissue heterogeneity in ischemic cardio-cerebrovascular disease

MIRI and CIRI can involve multiple forms of cell death during their pathological progression. Cardiac and cerebral tissues exhibit fundamental differences in structure, metabolic characteristics, and responses to ischemic insult. These inherent disparities contribute to distinct activation patterns of the cell death network comprising autophagy-dependent ferroptosis and PANoptosis in these two organs. Table 1 systematically compares and summarizes the relevant differences between MIRI and CIRI, providing a foundation for the following discussion. The subsequent sections will elaborate on the specific manifestations of these cell death pathways in each pathological context.

### 6.1 Myocardial ischemia-reperfusion injury

MIR acts as the pathophysiological basis of ischemic heart disease (Heusch, 2024) and serves as a critical phase following myocardial infarction during which blood flow is restored through revascularization strategies (Chapman et al., 2025). A hallmark feature of MIRI is irreversible cardiomyocyte death (Montecucco et al., 2016; Zhang et al., 2024). Recent investigations into MIRI have indicated that autophagy-dependent ferroptosis and PANoptosis synergistically drive cardiomyocyte injury, with PANoptosis exerting an amplifying effect on myocardial inflammation. As summarized in Table 1, autophagy-dependent ferroptosis acts as the initiating driver within the cell death network of MIRI. Ito and colleagues indicated in animal models that activation of NCOA4-mediated ferritinophagy in cardiomyocytes can specifically trigger ferroptosis, which may ultimately contribute to cardiomyocyte death and heart failure (Ito et al., 2021). Building upon this ferroptotic foundation, Zhang and colleagues indicated that in MIRI, ZBP1 is specifically upregulated in cardiomyocytes and assembles with RIPK3, caspase-8, and caspase-6 via its RHIM domain to form a non-canonical PANoptosome complex, thereby contributing to inflammatory amplification. Specific knockout of ZBP1 can effectively block PANoptosis-driven synergistic cardiomyocyte death, reduce infarct size, and improve cardiac function (Zhang Y. et al., 2025; Zhu L. et al., 2025). Luo and colleagues indicated that in a rat model of MIRI, individual inhibition of either apoptosis (using Z-vad) or ferroptosis (using Fer-1) conferred partial cardioprotection. However, combined inhibition of both death pathways produced significantly enhanced therapeutic efficacy, suggesting that apoptosis and ferroptosis synergistically drive cardiomyocyte death during MIRI pathogenesis (Luo et al., 2022). Wu and colleagues indicated that the LBH-CRYAB signaling axis simultaneously suppresses both cardiomyocyte apoptosis and ferroptosis in MIRI through the shared downstream effector p53, providing molecular-level evidence for the complexity of this regulatory network (Wu A. et al., 2024). p53 acts as a critical mechanistic link between autophagy-dependent ferroptosis and PANoptosis. Specifically, p53 can promote ferritinophagy to release labile iron, indirectly facilitating ferroptosis and inducing autophagy-dependent cell death. As an upstream regulatory node integrating multiple death signals, p53 is also implicated in PANoptosome regulation, coordinating NLRP3-caspase-1-GSDMD-mediated pyroptosis, RIPK3-MLKL-mediated necroptosis, and apoptosis. This integrative function mediates ROS burst and DNA damage, ultimately triggering PANoptosis and contributing to cardiomyocyte disarray (Tang L.-J. et al., 2021; Qu et al., 2024). Collectively, these findings indicate that inhibition of autophagy-dependent ferroptosis not only blocks iron supply and suppresses lipid peroxidation but also attenuates upstream signals that would otherwise activate PANoptosis. This dual effect generates synergistic protection, offering a promising combinatorial strategy for MIRI therapeutic intervention.

### 6.2 Cerebral ischemia-reperfusion injury

In contrast to MIRI, the cell death network in CIRI can exhibit greater complexity due to tissue heterogeneity. As summarized in Table 1, CIRI can involve multiple cell types, with PANoptosis predominantly occurring in glial cells-a pattern distinctly different from MIRI, which is primarily mediated by a single cell type, cardiomyocytes. Although no studies have directly demonstrated synergistic regulation between PANoptosis and autophagy-dependent ferroptosis in CIRI, core components of the PANoptosome are activated during autophagy-dependent ferroptosis, suggesting the existence of a highly integrated cell death network. Glial cell responses can represent a central feature of CIRI heterogeneity. Following injury stimulation, glial cells can engage cell death pathways centered around PANoptosis (She et al., 2023; Liu et al., 2022). Studies indicate that inhibition of the deubiquitinating enzyme USP13 may hyperactivate autophagy, thereby initiating cell death driven by the synergistic interaction of autophagy-dependent mechanisms, inflammation, and oxidative stress. During this process, increased reactive oxygen species (ROS) can activate the NLRP3/caspase-1/GSDMD-N pyroptosis pathway, further compromising membrane integrity and cellular homeostasis, indirectly facilitating a switch from pyroptosis to ferroptosis. Targeting this pathway may simultaneously alleviate both ferroptosis and inflammation, offering a therapeutic approach for CIRI. Regulatory nodes of PANoptosis in CIRI can exhibit considerable diversity. Beyond the classical RIPK3-MLKL pathway, PANoptosis also involves interactions with N-terminal kinases that participate in inflammatory signal transduction and contribute to necroptosis. Similarly, inactivation of TAK1 has been indicated to relieve inhibition of RIPK1 kinase activity, thereby contributing to neuronal necroptosis and apoptosis, and promoting PANoptosome formation (Naito et al., 2020; Malireddi et al., 2019). Furthermore, the thromboxane A synthase/thromboxane A2 signaling pathway has been indicated to simultaneously regulate both apoptosis and pyroptosis (Chueh et al., 2020). The NLRC4 nucleotide-binding oligomerization domain-like receptor inflammasome complex can also exhibit dual regulatory effects on these 2 cell death modalities (Ito et al., 2021; Wu A. et al., 2024). Therapeutically, targeting these shared nodes may demonstrate considerable potential. Existing studies have indicated that RIPK3 inhibitors reduce ischemic brain injury in preclinical disease models, with administration during the eperfusion phase exerting protective effects on the brain through mechanisms including HIF-1α inhibition (Karki et al., 2021b; Tang L.-J. et al., 2021). Ginsenoside Rb3 has been indicated to concurrently influence inflammation and ferroptosis via the NLRP3/NF-κB/GPX4 pathway, achieving multi-target neuroprotection and reducing injury following middle cerebral artery occlusion/reperfusion (Xie et al., 2025). As summarized in Table 1, potential therapeutic strategies for CIRI include targeting upstream regulatory molecules such as TAK1 or USP13, thereby intervening at shared nodes within the death network rather than modulating individual pathways. She and colleagues reviewed the role of mitochondria in inducing PANoptosis and ferroptosis in CIRI (She et al., 2023). Fan and colleagues, through both *in vitro* and *in vivo* experiments targeting the GATA6/miR-193b/ATG7 axis, indicated that regulating autophagy and ferroptosis attenuates CIRI (Fan et al., 2023). The protective effect of cGAS-STING inhibition in CIRI has been demonstrated in both *in vivo* and *in vitro* models. Zhang et al. reported that this intervention suppresses NLRP3 inflammasome activation, highlighting a key regulatory node connecting innate immunity to cell death (Zhang X. et al., 2025). Although direct evidence requires further exploration, targeting upstream hubs where autophagy-dependent ferroptosis and PANoptosis intersect-such as USP13, TAK1, and RIPK3-may enable simultaneous intervention in both inflammation and oxidative stress, thereby synergistically blocking multiple cell death pathways. These nodes represent critical targets for overcoming CIRI progression. This conceptual framework suggests that concurrently targeting upstream hubs of multiple death pathways may yield more effective neuroprotective outcomes.

## 7 Discussion and conclusion

Previous studies have indicated the independent roles of autophagy-dependent ferroptosis and PANoptosis in ischemia-reperfusion injury (IRI). Autophagy-dependent ferroptosis can be driven by multiple selective autophagic mechanisms: NCOA4-mediated ferritinophagy releases labile iron to initiate Fenton chemistry; lipophagy supplies polyunsaturated fatty acids as substrates for lipid peroxidation; and selective degradation of defensive proteins such as GPX4 compromises cellular antioxidant capacity. Collectively, these processes may constitute the molecular foundation of actively driven ferroptosis, enriching our understanding of regulated cell death mechanisms. PANoptosis can represent a distinct inflammatory programmed cell death phenotype characterized by the concurrent and interdependent occurrence of molecular events associated with pyroptosis, apoptosis, and necroptosis. The PANoptosome has been indicated as the central molecular platform executing this death modality, integrating signaling components from all three pathways. Notably, when one pathway becomes restricted, compensatory activation of alternative pathways may ensure the execution of inflammatory cell death. These findings may provide a foundation for understanding the regulatory mechanisms of cell death in IRI.

### 7.1 Synergistic network of autophagy-dependent ferroptosis and PANoptosis

Building upon the independent mechanisms described above, recent studies suggest the existence of more complex interactions between these 2 cell death modalities. Based on available evidence, we propose an integrative framework: in myocardial and cerebral ischemia-reperfusion injury (IRI), autophagy-dependent ferroptosis and PANoptosis may constitute a synergistically amplified cell death network through shared key molecular hubs. The dual characteristics of autophagy-dependent ferroptosis-metabolic dysregulation coupled with oxidative damage-catalyze inflammatory amplification effects, forming a death network where reactive oxygen species (ROS), iron metabolism, and lipid metabolism intersect and synergize. PANoptosis exhibits prominent “inflammatory programmed” cell death features, combining key molecules from multiple death pathways with robust inflammatory responses. Both modalities share core characteristics of “multi-pathway crosstalk” and “inflammatory exacerbation.” Therefore, we propose constructing a conceptual cell death network integrating these two novel death modalities to address IRI from multiple dimensions and potentially reverse cellular damage. As common upstream triggers, oxidative stress, mitochondrial dysfunction, and inflammatory responses elicited by IRI can simultaneously activate initiation programs for both autophagy-dependent ferroptosis (via NCOA4 upregulation and lipophagy) and PANoptosis (via ZBP1 sensing and NLRP3 activation). Shared molecular hubs-including STING, NCOA4, GPX4, RIPK1, and NLRP3-can participate in the crosstalk between these two death modalities. ROS and lipid peroxides serve dual functions: they are both products and executors of autophagy-dependent ferroptosis, while also functioning as critical signals that activate the NLRP3 inflammasome and drive PANoptosome assembly. NLRP3 itself acts as a connecting node, receiving activating signals from the ferroptosis pathway while serving as a core component of the PANoptosome to initiate inflammatory cell death. RIPK1/RIPK3 not only participate in necroptosis execution but also feed back to regulate autophagic activity and GPX4 expression, establishing positive feedback amplification loops. Membrane rupture resulting from ferroptosis releases damage-associated molecular patterns (DAMPs), further amplifying inflammatory responses and exacerbating PANoptosis. Conversely, inflammatory cytokines released during PANoptosis intensify oxidative stress, reciprocally promoting ferroptosis. This reciprocal amplification may represent a key mechanism accelerating cell death in IRI. As illustrated in Table 1, tissue heterogeneity manifests in distinct activation patterns of these death networks. In MIRI, the high metabolic demand and heightened oxidative stress sensitivity of cardiomyocytes position autophagy-dependent ferroptosis as the initiating driver, subsequently activating PANoptosis to amplify inflammatory responses and exacerbate cardiomyocyte death. In CIRI, cellular complexity generates a multifocal pattern: neurons may be more susceptible to ferroptosis, while glial cells predominantly undergo inflammatory cell death via PANoptosis, with reciprocal interactions mediated by signaling molecules within the microenvironment. Additionally, brain-specific features, including glutamate excitotoxicity and the blood-brain barrier, introduce unique regulatory dimensions.

### 7.2 Therapeutic implication

Traditional therapeutic approaches have largely focused on targeting single-cell death pathways, such as inhibiting ferroptosis or blocking apoptosis individually. However, these strategies have demonstrated limited efficacy in experimental studies. Therefore, targeting shared hubs within the interactive death network rather than individual pathways may represent a more effective therapeutic paradigm. This strategic shift from targeting single pathways to intervening in interactive networks warrants further exploration. Several promising approaches have emerged. Direct targeting of core interactive nodes, including NLRP3 inhibitors, RIPK1 inhibitors, and STING inhibitors, simultaneously modulates multiple death pathways and has demonstrated protective effects in both MIRI and CIRI models. Combined blockade of complementary pathways, such as inhibiting ferroptosis with ferrostatin-1 or deferoxamine (DFO) together with PANoptosis inhibition via caspase blockade, may simultaneously interrupt both death network initiation and inflammatory amplification, yielding synergistic protection. Multi-target natural products, including active components of traditional Chinese medicine such as icariin and ginsenoside Rb3, have been shown to concurrently regulate molecules involved in both autophagy-dependent ferroptosis and PANoptosis, demonstrating potential for multi-target intervention. However, as summarized in Table 1, important differences exist between cardiac and cerebral IRI that impact therapeutic translation, including the influence of the blood-brain barrier on drug delivery, distinct therapeutic time windows, and predominant death modalities. These tissue-specific factors require consideration in therapeutic development. The intervention strategies discussed above may offer new insights for treating other ischemia-related diseases, potentially improving patient outcomes and survival rates. Nevertheless, the impact of tissue heterogeneity on therapeutic efficacy necessitates organ-specific optimization of treatment approaches built upon a common mechanistic framework.

### 7.3 Limitations and future perspectives

Although this review proposes a synergistic regulatory network between autophagy-dependent ferroptosis and PANoptosis, several limitations in current research should be acknowledged. These limitations may also delineate directions for future investigation. First, limitations in experimental evidence. The mechanisms underlying the interaction between these two death modalities are predominantly derived from *in vitro* cell models. Critical interactive nodes, such as ROS-mediated NLRP3 activation and lipid peroxide-facilitated PANoptosome assembly, have been primarily validated through cellular experiments. The core molecular mechanisms discussed in Sections 4.1 through 4.3 are also largely based on *in vitro* studies. Although a limited number of animal model studies (e.g., ZBP1 in MIRI; NCOA4 and STING in CIRI) have provided preliminary *in vivo* evidence supporting this network, overall evidence directly demonstrating the *in vivo* interaction between autophagy-dependent ferroptosis and PANoptosis remains insufficient. Most conclusions are derived from logical integration and inference based on independent pathway studies, and systematic validation of dynamic changes in intact animal models is lacking. Furthermore, the discussion of cardiac and cerebral tissue heterogeneity in Section 5 lacks direct evidence from patient samples. Clinical studies investigating autophagy-dependent ferroptosis or PANoptosis in IRI patients remain absent. Future research should utilize clinical patient samples to validate the expression of key molecules and explore multiplex biomarkers capable of simultaneously monitoring multiple death modalities. Second, the dynamic nature of the network remains unknown. The interaction between autophagy-dependent ferroptosis and PANoptosis likely exhibits dynamic changes across different phases of IRI. The ischemic phase may be characterized by metabolic disruption and ferroptosis initiation; early reperfusion by oxidative stress burst and inflammasome activation; and the late phase by PANoptosis-mediated inflammatory amplification. Integration of multi-omics approaches with spatiotemporal analysis may reveal critical regulatory windows for therapeutic intervention. Finally, the most critical challenge: clinical translation. Although targeting interactive nodes has demonstrated protective effects in animal models, multiple obstacles impede clinical translation. Tissue heterogeneity between the heart and brain may necessitate organ-specific delivery strategies. Simultaneous intervention in multiple pathways may engender unforeseen adverse effects. The absence of biomarkers capable of monitoring multiple death modalities complicates clinical efficacy assessment. Future efforts should focus on developing multi-target drugs, exploring optimized combination regimens, and establishing surrogate markers correlated with clinical endpoints.

### 7.4 Conclusion

In summary, autophagy-dependent ferroptosis and PANoptosis do not function as isolated events in MIRI and CIRI. Rather, they constitute a synergistically amplified cell death network through shared molecular hubs. This conceptual framework provides a novel perspective for understanding the complex pathophysiology of IRI and establishes a theoretical foundation for developing combination therapeutic strategies targeting interactive nodes within multiple cell death pathways. However, it must be acknowledged that the key evidence supporting this network remains predominantly derived from *in vitro* studies. Animal model evidence remains insufficient, and clinical studies are entirely lacking. The precise dynamics of this network *in vivo* and its clinical translational value require further validation. Future research should focus on several key directions: utilizing conditional gene knockout animal models to directly validate the role of critical interactive nodes in IRI; conducting clinical sample analyses to explore the potential of key molecules as prognostic biomarkers or therapeutic targets; elucidating the spatiotemporal dynamics of the network to identify critical intervention windows; and developing multi-target intervention strategies. Ultimately, these efforts aim to achieve a paradigm shift in therapeutic strategy from targeting single pathways to modulating synergistic networks.
